# Titan cell formation is unique to *Cryptococcus* species complex

**DOI:** 10.1080/21505594.2020.1772657

**Published:** 2020-06-04

**Authors:** Mariusz Dyląg, Rodney J. Colon-Reyes, Lukasz Kozubowski

**Affiliations:** Department of Genetics and Biochemistry, Eukaryotic Pathogens Innovation Center, Clemson University, Clemson, SC, USA

**Keywords:** Fungi, virulence factors, pathogenicity, cryptococcosis, morphological transition

## Abstract

Members of the *Cryptococcus* species complex stand out by unique virulence factors that allowed evolutionary transition to pathogenesis. Among the factors contributing to cryptococcosis is a morphological transformation into giant (Titan) cells. It remains unclear whether species outside of the *C. neoformans*/*C. gattii* species complex are capable of titanization. We utilized two recently developed protocols that allow obtaining Titan cells *in vitro* to test if titanization occurs in non-*C. neoformans*/*C. gattii* species. We find that none of the tested strains, representing 10 species of basidiomycetous yeasts and the ascomycetous yeast *Saccharomyces cerevisiae*, undergo significant titanization under conditions that promote robust Titan cell formation in *C. neoformans*/*C. gattii* species complex. *C. terreus* formed occasional enlarged cells through a mechanism potentially similar to that of titanization. Our findings suggest that titanization is a rare phenomenon among basidiomycetous yeasts that occurs mostly in members of the *C. neoformans/C. gattii* species complex.

## Introduction

Basidiomycetous yeasts are an ecologically heterogeneous group of fungi that includes both saprophytic as well as pathogenic species. A significant fraction of the latter group comprises of human and animal pathogens, including such genera as *Cryptococcus, Malassezia, Rhodotorula,* and *Trichosporon* [1]. In particular, members of the *Cryptococcus neoformans* and *Cryptococcus gattii* species complex are the etiological agents of fatal systemic mycoses. *C. neoformans* species complex includes *C. neoformans* var. *grubii* (serotype A, currently considered a separate species named *C. neoformans*) and *C. neoformans* var. *neoformans* (serotype D, currently considered separate species named *C. deneoformans*) [2,3]. *C. gattii* species complex includes five genetically distinct groups that have been recently recognized as separate species (VGI, *C. gattii*; VGII, *C. deuterogattii*; VGIII, *C. bacillisporus*; VGIV, *C. tetragattii*; VGIV/VGIIIc, *C. decagattii*) [2,3]. In recent years, nearly one million cases of cryptococcal meningitis occur annually resulting in over 600,000 deaths globally. Moreover, cryptococcosis is responsible for 15–17% AIDS-related deaths on a global scale [4,5]. While *C. neoformans* can cause mainly opportunistic infections in immunocompromised patients, representatives of the *C. gattii* species complex are capable of infecting also immunocompetent individuals [6–9]. Moreover, it is well established that *C. neoformans* is responsible for the majority of cryptococcal infections, while *C. deneoformans* is less common [8]. Among *Cryptococcus* species outside of the *C. neoformans*/*C. gattii* species complex, the following species have been described as causing occasional infections in humans: *C. laurentii* [10–13], *C. albidus* [10,14–16], *C. curvatus* [17,18], *C. uniguttulatus* [19], and *C. adeliensis* [20]. Such casuistic infections, reviewed in literature most recently by Smith et al. [21] can be also systemic in case of strains able to grow at 37°C. What makes those selected *Cryptococcus* species capable of infecting humans is an important question, the answer to which remains incomplete. Among the best-described cryptococcal characteristics necessary for pathogenicity are the ability to proliferate at 37°C, melanisation, formation of capsule, and the capability of hydrolyzing urea [9,22]. Most of the above-mentioned “virulence factors” can be observed together only in representatives of the *C. neoformans*/*C. gattii* species complex, what could potentially explain the basis of their evolutionary success as pathogens.

Dimorphism is one of the common features of human fungal pathogens [23,24]. A well-documented example of a dimorphic switching critical for pathogenicity is yeast to hypha transition characteristic for dimorphic fungi clustered within five genera [23,24]. Morphological transition of *C. neoformans/C. gattii species complex* to form enlarged cells termed Titan cells is a particularly striking manifestation of a perfect adaptation to evade the mammalian immune system and enhance dissemination in the host [25]. Typically *C. neoformans* cells range in size from 4 to 6 µm, whereas the size larger than 10 µm has been considered as indicative of titanization. Other key features of Titan cells are thicker cell wall, single large vacuole and increased ploidy within a single nucleus [26–28]. Furthermore, Titans can produce daughters of “normal” size, which can be haploid or frequently aneuploid [29]. Titan cells are resistant to phagocytosis due to the enlargement of the cell body [25,26,30,31]. Moreover, it has been demonstrated that Titan cells and aneuploid daughter cells of Titans show increased resistance to many physico-chemical factors and toward some drugs commonly used in the therapy of cryptococcosis [29]. However, titanization may not be required for pathogenicity as some clinical isolates of *C. neoformans* are presumably not capable of undergoing this morphological transition [32–34]. Therefore, it remains unclear to what extent titanization is important for human cryptococcosis and whether this striking characteristic is unique to pathogenic *Cryptococcus* species, especially since titanization has never been studied in species outside of the *C. neoformans*/*C. gattii* species complex.

Recent investigations led to considerable advances in our understanding of the nature of titanization, by uncovering external stimuli that are sufficient and genes that are essential for this morphological transition [32–34]. Two well-documented pathways involved in titanization are the cAMP-mediated signaling (dependent on Gpr4/Gpr5 receptors, adenylyl cyclase Cac1, Pka1 kinase, and the transcription factor Rim101) and the mating pathway [27,35,36]. Until recently the progress in identifying more regulators has been hampered by the lack of a suitable *in vitro* system. However, recently published work from several laboratories delivered new opportunities towards a thorough understanding of titanization by developing *in vitro* conditions capable of stimulating this morphological change [32–34]. This breakthrough research led to the identification of novel positive (Gat201, Ada2, Cap59, Cap60, Ric8, Sgf29, Lmp1) and negative (Usv101, Pkr1, Tsp2) regulators that were important for titanization under specific conditions [32,34]. These studies also led to an overarching conclusion that titanization can be stimulated by a variety of external signals, including CO_2_, hypoxia, exposure to serum (specifically two serum components, phospholipids, and bacterial peptidoglycan), and quorum sensing [32–34]. Importantly, these novel protocols to induce titanization *in vitro* have provided an opportunity to test to what extent is the ability to form Titans conserved in other *Cryptococcus* species.

Based on a survey of 23 strains that represent 10 basidiomycetous yeast species outside of the *C. neoformans*/*C. gattii* species complex, we postulate that *C. neoformans/C. gattii* species complex is unique among other basidiomycetous yeasts with regard to the ability to form *bona fide* Titan cells.

## Materials and methods

### Tested strains and media

All the strains used in this study are listed in Table S1 (Appendix 1), which includes information about the origin (source), place of primary isolation, and actual taxonomic position for each tested strain. Strains were routinely cultured on YPD (Yeast extract Peptone Dextrose) semi-solid medium, followed by liquid medium: 1% yeast extract, 2% bacto-peptone and in case of semi-solid media 2% bacto-agar (BD Difco, Sparks, MD, USA, cat. no. REF212720, REF211820, and REF212720) and filter-sterilized 2% glucose (VWR International LLC, West Chester, PA, USA, cat. no. BDH0230). In case of Titan induction experiment, cells were grown overnight in 5 ml YNB (Yeast Nitrogen Base) liquid medium: 0.67% Yeast Nitrogen Base with amino acids, pH 5.5 (Sigma cat. no. Y1250, St. Louis, MO, USA), 2% glucose at 30°C with horizontal shaking at 220 rpm (Thermo Scientific, MAXQ4450).

### In vitro *Titan cells induction*

Titan cells were generated *in vitro* according to two recently described protocols [33,34]. To follow the protocol by Dambuza et al. [33], experiments were performed in sterile, non-heat inactivated 10% Fetal Bovine Serum (FBS, Sigma, cat. no. F6178) diluted in 1× concentrated PBS (Dulbecco`s Phosphate-Buffered Saline w/o Ca^2+^ and Mg^2+^, cat. no. REF21-031-CV, Corning cellgro®, Manassas, VA, USA), at final pH equal to 7.4. Original concentrated FBS was normally stored in 2.5 ml aliquots at −20°C to prevent the repeated freeze-thaw procedure. Cells were incubated in static conditions for 48 h or 120 h at 37 or 30°C under 5% CO_2_ atmosphere (New Brunswick an Eppendorf company, Galaxy 170 S, Ayrshire, Scotland). To follow the protocol according to Hommel et al. [34], cells were initially incubated in 10 ml of the YPD liquid medium at 30°C with horizontal shaking at 150 rpm (Thermo Scientific, MAXQ4450) for 22 h. Subsequently, cultures were centrifuged (5 min, 3,000 rpm, room temperature) and the cells were washed twice using sterile liquid MM medium: 15 mM D-glucose, 10 mM MgSO_4_, 29.4 mM KH_2_PO_4_, 13 mM Glycine and 3.0 µM Thiamine (Sigma cat. no. G8270, M7506, P5655, G7126 and T4625, respectively). Finally, cells were resuspended in 1 ml of MM medium in 1.5 ml Eppendorf tubes to achieve density equal to 10^4^ cells/ml (based on calculations in haemocytometer). Cell suspensions prepared in this way were incubated for 5 days at 30°C with horizontal shaking at 800 rpm (Eppendorf Thermomixer, Eppendorf, Poland).

### Evaluation of cell viability

Viability of cells was evaluated qualitatively by examination of micro-wells of the titration plate directly under the microscope and quantitatively by plating. For this purpose, 100 µl volume from each micro-well of the titration plate was collected (after mixing) and either initially diluted if necessary using sterile 1× concentrated PBS or directly spread on YPD semi-solid medium. Plates were incubated 48 h at 30°C in the incubator w/o CO_2_ (Thermo Scientific, Heratherm Incubator IMH180, Langenselbold, Germany). After this time plates were photographed and colony counting was performed.

### Negative staining with nigrosin

For polysaccharide capsule visualization, negative staining technique with 10% nigrosin solution (nigrosin 10%, formalin 0.5% in water) was used [37]. This method permits visualization of the transparent and unstainable capsule in the case of *Cryptococcus* spp. For this purpose 5 µl of nigrosin solution and 5 µl of dense yeast liquid culture was mixed on the surface of the microscopic slide and covered with a thin cover glass. Microscopic preparations were examined using immersion oil and magnification 1000× under the light microscope (OPTA-TECH, model: MB200) with a built-in camera for photographic documentation (OPTA-TECH, 3MP) and interfaced with OptaView-IS software (OptaView-IS, version 3.6.6).

### Microscopy and imaging

Microscopic observations were performed under the Axiovert 200M microscope using either the 20× or 100× objective (ID#M 202086, Carl Zeiss MicroImaging, Inc., Thornwood, NY, USA) with a built-in camera for photographic documentation (AxioCam HRm) and interfaced with AxioVision Rel 4.8 software (Carl Zeiss, Thornwood, NY). For evaluation of cell body diameter at least 100 cells for each treatment were measured based on the images using ImageJ (https://imagej.nih.gov/ij/) and the data were plotted using the GraphPad Prism v. 8 software.

### Construction of the phylogenetic tree

**Figure 1.**
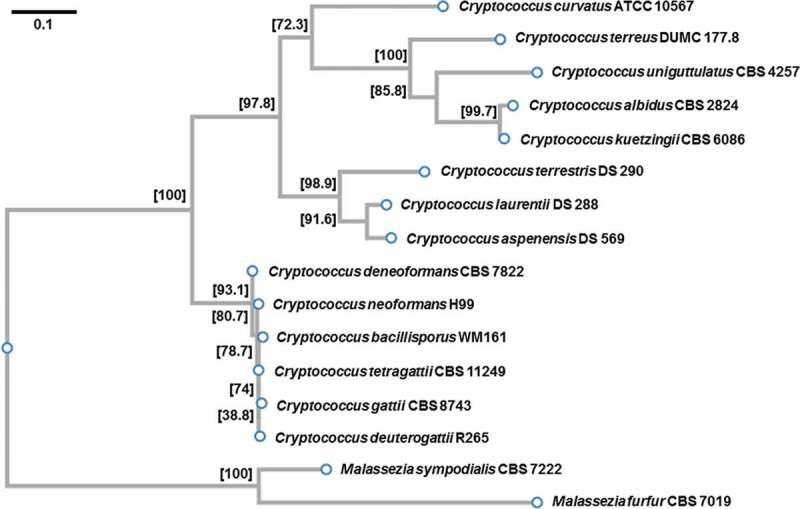
Phylogenetic tree illustrating the relatedness of the species included in this study. Values at nodes are SH-like local support. Scale bar indicates one substitution per 10 nucleotide positions.

The multiple sequence alignment of the nuclear ribosomal internal transcribed spacer (ITS) region was performed based on CLUSTALW software as implemented on the GenomeNet (https://www.genome.jp/tools-bin/clustalw). All the sequences (see Appendix 1) used to construct a phylogenetic tree were obtained from the GenBank of the National Center for Biotechnology Information (NCBI, Bethesda, MD, USA). Alignment and phylogenetic reconstructions were performed using the function “build” of ETE3 v3.1.1 [38]. The Phylogenetic tree (Figure 1) was constructed using fasttree with slow NNI and MLACC = 3 [39]. Values at nodes are SH-like local support. Scale bar indicates one substitution per 10 nucleotide positions.

### Statistical analysis

To test differences between the median of cell body diameter, first the Normality Kolmogorov-Smirnov test was performed and subsequently the Mann Whitney and Kluskal-Wallis tests were performed with the use of GraphPad Prism v. 8 software.

## Results

### Phylogenetic relatedness of the tested species

We tested the following eight non-*neoformans*/non-*gattii Cryptococcus* species for their ability to undergo titanization *in vitro: C. albidus* (2 strains), *C. aspenensis* (4 strains), *C. curvatus* (2 strains), *C. kuetzingii* (1 strain), *C. laurentii* (5 strains), *C. terrestris* (4 strains), *C. terreus* (1 strain), and *C. uniguttulatus* (two strains). We also tested representative strains from the *C. gattii* species complex (seven strains). To include other basidiomycetous yeasts outside of the *Cryptococcus* genus, we tested two species of *Malassezia: M. furfur* (1 strain) and *M. sympodialis* (1 strain). We also included one reference strain of *Saccharomyces cerevisiae* as a representative of ascomycetous yeasts. All strains utilized in this study are listed in Table S1 (Appendix 1).

We searched existing databases for sequences of the nuclear ribosomal internal transcribed spacer (ITS) regions to construct a phylogenetic tree illustrating relatedness of the species represented by strains tested in our study (Figure 1) [40]. It should be underlined that most of the basidiomycetous yeasts tested here represent species more or less phylogenetically related to the genus *Filobasidiella* Kwon-Chung (1976) including the most important species (in the context of the prevalence of cryptococcosis): *C. neoformans* and representatives of *C. gattii* species complex [8]. According to recently updated taxonomy of Tremellomycetes [41], strains used in this study belong to the following genera: Cutaneotrichosporon (*C. curvatus*), Filobasidiella (*C. gattii* and *C. neoformans*), Filobasidium (*C. uniguttulatus*), Naganishia (*C. albidus* and *C. kuetzingii*), Solicoccozyma (*C. terreus*), Papiliotrema (*C. aspenensis, C. laurentii,* and *C. terrestris*). While many of the species included here were originally given the name *Cryptococcus*, the phylogenetic analysis suggested a significant divergence (Figure 1). Thus, our study included species representing considerably diverse groups among basidiomycetous yeasts, an important aspect given that occasionally even closely related fungal strains reveal significant genetic divergence [42].

### Assessment of the influence of the temperature on titanization

Three *in vitro* protocols to induce the formation of *bona fide* Titan cells in *C. neoformans* have been published recently [32–34]. In all three cases putative Titan cells have formed that met established criteria characteristic of cell gigantism observed originally *in vivo* during murine infection caused by *C. neoformans* [26,27]. While the titanization protocol developed by Hommel et al. recommended incubation at 30°C, the methods established by Dambuza et al. and Trevijano-Contador et al. suggested incubation at 37°C [32–34]. Therefore, we first analyzed how temperature affected the growth of the strains included in our study. We reasoned that the ideal temperature for titanization is close to but not above the maximum temperature a given strain tolerates. While *C. neoformans* and *C. deuterogattii* were capable of proliferating at 37°C, as reported previously, the majority of non-*neoformans*/non-*gattii* species could not grow at this temperature (Table S1). Based on the fact that the majority of these strains could not proliferate at 37°C and grew at 30°C, we decided to utilize 30°C when testing titanization of these species.

**Figure 2.**
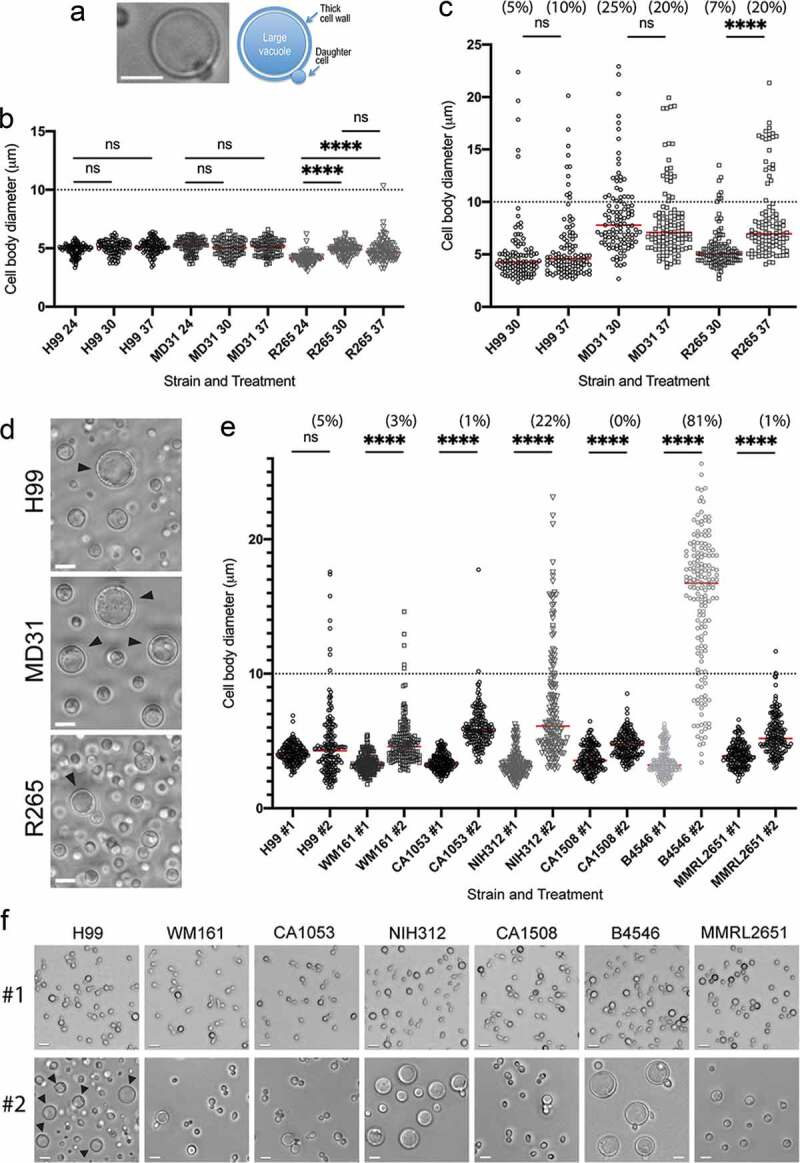
Titan cell formation among the *C. neoformans* and *C. gattii* species complex.

We assessed the ability to form Titan cells based on measurements of the cell diameter and a visual inspection of cells after incubation under titanization conditions. Specifically, we defined Titan cells as cells with diameter of close to, or larger than 10 µm, with characteristic central single large vacuole, and a thick cell wall as judged based on differential interference contrast (DIC) microscopy (Figure 2(a)). As we decided to test Titan cell formation at 30°C, it was important to assess whether lowering the temperature to 30°C affects titanization in the protocol introduced by Dambuza et al. [33]. First, we tested the effect of the temperature on the cell size when *C. neoformans* (strains H99 and MD31) and *C. deuterogattii* (strain R265) were grown in a standard medium utilized for the growth of *C. neoformans* (YPD medium) (Figure 2(b)). The diameters of cells of all three strains were no larger than 7 microns at 24 and 30°C. At 37°C, cell diameters of H99 and MD31 remained under 7 microns and 2% of cells of R265 (two cells out of 100 measured) had diameters between 7 and 11 microns. Although for the R265 there was a modest increase in cell diameter between 24°C and either 30 or 37°C, a change in temperature from 30 to 37°C did not influence cell diameter significantly for any of the strains. Next, we subjected all three strains to titanization conditions according to Dambuza et al., at either originally reported 37°C or at 30°C, to test if lowering the temperature to 30°C had a significant effect on the formation of Titans. Consistent with previous findings, *C. neoformans* serotype A strains H99 and MD31, and *C. deuterogattii* clinical isolate, strain R265, formed Titan cells after 48 hours of incubation in 10% fetal bovine serum (FBS) under 5% CO_2_ at 37°C (Figure 2(c)) [32,33]. Notably for strain R265, the percentage of Titan cells and the average size of cells were higher at 37°C as compared to 30°C, in contrast to strains H99 and MD31 for which temperature had a lesser effect on titanization (Figure 2(c)). Furthermore, strain R265 did not proliferate significantly within 48 hours under titanization conditions, as the number of cells only increased 3 times at 37°C and 5 times at 30°C, in contrast to *C. neoformans* H99 and MD31 for which an increase in cell number was between ~120 and 500 times (Table 1). Thus, host temperature was not essential for titanization of strains H99, DM31, and R265 (Figure 2(c, d)). However, the average size of Titan cells was smaller for R265 strain at 30°C as compared to 37°C suggesting that the temperature of 37°C potentiates this process in this particular strain (Figure 2(c, d)). In contrast, neither H99 nor MD31 exhibited a significant difference in cell diameter between 30 and 37°C under titanization conditions according to Dambuza et al. These findings confirmed that titanization can occur at 30°C but 37°C may further stimulate this morphological transition at least in some species/strains. We also tested titanization at 30°C of additional 6 representative strains of the *C. gattii* species complex (5 strains of *C. bacillisporus* and 1 strain of *C. tetragattii*). Although cells of all 6 tested strains became larger at titanization conditions (indicated in Figure 2(e, f) as #2), as compared to control conditions (indicated in Figure 2(e, f) as #1), the ability to form Titans varied drastically among these isolates (Figure 2(e, f)). Notably, *C. bacillisporus* strain B4546 exhibited particularly robust titanization with ~81% of cells (136 out of 167 measured) with diameter above 10 μm and an average diameter of ~15 μm, contrasting the average cell diameter of the YPD-grown B4546 cells (indicated in Figure 2(e, f) as #1) of ~4 μm. Thus, at least some strains of the *C. neoformans*/*C. gattii* species complex are capable of titanization at conditions described by Dambuza et al. at both 30 and 37°C, although the degree of titanization (percentage of Titans and the average size of Titans) varies between strains and for some strains is higher at 37 as compared to 30°C.

**Table 1.** Total number of cells and percentage of Titan cells after 48 h incubation under titanization conditions by Dambuza et al., at 30°C. The initial inoculum was 10^3^ cells.

Strain	Cell number (% Titans) after 48 h incubation
*C. deuterogattii*, R265	5.4 × 10^3^ (12.6%)
*C. neoformans*, H99	2.2 × 10^5^ (7.2%)
*C. neoformans*, MD31	1.2 × 10^5^ (8.4%)
*C. aspenensis*, DS573	1.2 × 10^6^ (0%)
*C. aspenensis*, DS572	3.2 × 10^6^ (0%)
*C. aspenensis*, DS570	9.4 × 10^5^ (0%)
*C. aspenensis*, DS569	1.8 × 10^6^ (0%)
*C. kuetzingii*, MUCL27749	1.3 × 10^5^ (0%)
*C. laurentii*, DS620	1.2 × 10^6^ (0%)
*C. laurentii*, DS386	3.8 × 10^6^ (0%)
*C. laurentii*, DS619	2.3 × 10^6^ (0%)
*C. laurentii*, DS621	3.2 × 10^7^ (0%)
*C. laurentii*, DS288	5.6 × 10^7^ (0%)
*C. uniguttulatus*, MD29	8.3 × 10^4^ (0%)
*C. uniguttulatus*, MD26	8.6 × 10^3^ (0%)
*C. albidus*, MD22	7.6 × 10^5^ (0%)
*C. albidus*, MD41	1.2 × 10^6^ (0%)
*C. terrestris*, DS291	1.2 × 10^6^ (0%)
*C. terrestris*, DS233	3.9 × 10^5^ (0%)
*C. terrestris*, DS234	2.4 × 10^6^ (0%)
*C. terrestris*, DS290	1.4 × 10^7^ (0%)
*C. terreus*, DUMC177.8	1.1 × 10^3^ (0%)
*C. curvatus*, MD110/2009	1.5 × 10^6^ (0%)
*C. curvatus*, MD120/2009	1.2 × 10^7^ (0%)
*S. cerevisiae*, BY4741	5.4 × 10^5^ (0%)
*M. furfur*, CBS 7019	1.3 × 10^4^ (0%)
*M. sympodialis*, ATCC 42132	2.4 × 10^4^ (0%)

(a) Features of a Titan cell as observed with the DIC microscopy. *C. neoformans* var. *grubii* (strain H99) was grown under titanization conditions according to Dambuza et al. [33] at 30°C for 5 days. (b) The effect of temperature (24, 30, and 37°C) on cell diameter of two *C. neoformans* strains (H99 and MD31) and *C. deuterogattii* (R265). Cells were incubated in YPD for 24 hours prior to imaging. (c) The effect of 37°C as compared to 30°C on the median cell diameter under titanization conditions [33] for *C. neoformans* (H99, MD31) and *C. deuterogattii* (R265). (d) Representative images illustrating cells incubated at titanization conditions [33] at 30°C, whose diameters are plotted in C. (e) The effect of incubation under titanization conditions [33] at 30°C for 5 days (marked as #2) on cell body diameter of *C. neoformans* (H99) and representative strains of the *C. gattii* species complex. Cells were grown in YPD at 30°C for 8 hours as control (marked as #1). (F) Images of cells whose diameters are plotted in E (#1 – YPD control, #2 – titanization conditions at 30°C for 5 days). The number of cells counted for each condition were 100 (in b), 110 (;in c), and 167 (in e). The numbers above each plot in C and E represent the percentage of cells with the diameter above 10 μm. Statistical significance of the difference in the mean cell diameter is indicated as follows: ns (not significant), **** (p < 0.0001) Bars in (a, d), and (f) represent 10 µm.

### C. neoformans/C. gattii *species complex is unique in the ability to undergo titanization*

**Figure 3.**
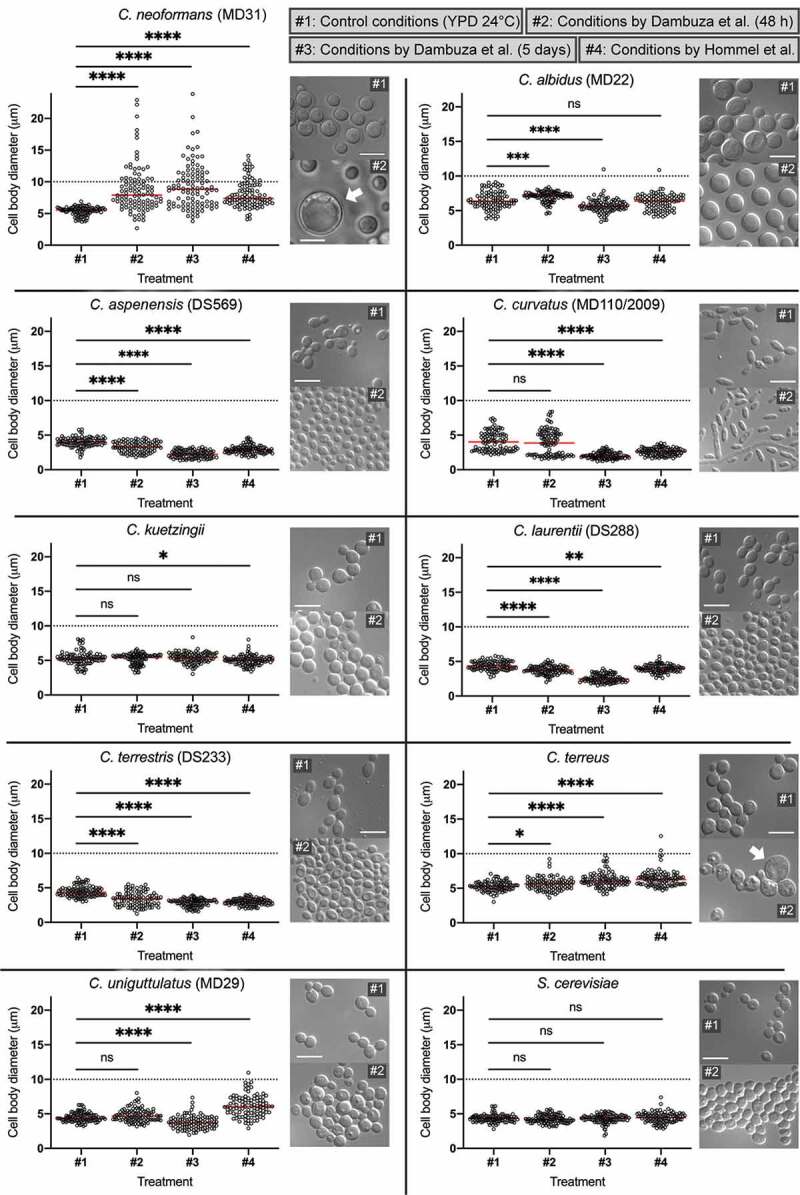
Members of the *Cryptococcus* species complex are unique in their ability to form Titan cells.

Most of the non-*neoformans*/non-*gattii Cryptococcus* strains, when subjected to titanization conditions according to Dambuza et al. with incubation temperature set at 30°C (indicated in Figure 3 as #2), have proliferated significantly, as the number of cells has increased at least 1000 times within 48 hours of incubation (Table 1). The exception was a relatively slower growing *C. kuetzingii* (130 times increase in cell number), the two strains of *C. uniguttulatus* (~9 times, and ~80 times increase), and nearly completely inhibited *C. terreus* (Table 1). The two *Malassezia* species grew poorly under these conditions (~10 times increase in cell number) and *S. cerevisiae* proliferated significantly (540 times increase in cell number).

*C. neoformans* (MD31), and representatives of other species, as indicated, were grown in YPD control medium at 24°C (indicated as #1), subject to the *in vitro* titanization protocol established by Dambuza et al. [33] with a modified temperature to 30°C and incubated for 48 h (indicated as #2) or 5 days (indicated as #3) or subject to conditions developed by Hommel et al. [34] (indicated as #4). Cell diameters of 100 cells from each condition were measured and plotted. For each strain, representative images are shown of the cells grown at 24°C in rich YPD medium (#1) or after 48-h incubation in titanization conditions by Dambuza et el. at 30°C (#2). Statistical significance of the difference in the mean cell diameter is indicated as follows: ns (not significant), * (0.05 > p < 0.01), ** (0.01 > p < 0.001), *** (0.001 > p < 0.0001), **** (p < 0.0001). Bars represent 10 µm.

Strikingly, after 48 hours at 30°C none of the non-*neoformans*/non-*gattii* strains formed cells that could be classified as *bona fide* Titans according to previously established criteria (Figure 3, Table 1, and Figure S2(a)). We measured cell diameter for selected strains of each species to assess cell enlargement under titanization conditions. The mean cell diameter of three species, *C. albidus* (MD22), *M. furfur*, and *C. terreus* was larger at titanization conditions (#2) as compared to cultures grown in control conditions (#1), although no cells were larger than 10 microns (Figure 3, Figure S2(a)). Interestingly, average cell diameters of three species, *C. aspenensis* (DS569), *C. laurentii* (DS288), and *C. terrestris* (DS233) were smaller as compared to those of cells grown in control conditions (Figure 3). Average cell diameters of the remaining four evaluated species were not significantly different compared to the control (Figure 3).

We observed morphological differences among the non-*neoformans*/non-*gattii* species under “titanization” conditions according to Dambuza et al. after 48 h of incubation (Figure 3). Most of *C. albidus* cells, uniquely among other species, became uniformly round and unbudded and the cells were arranged with spaces between, indicative of possible capsule formation. *C. terrestris* and to a lesser extent also *C. aspenensis* were the only species that exhibited a large proportion of cells with a single enlarged vacuole. In four species (*C. laurentii, C. terreus, C. uniguttulatus*, and *S. cerevisiae*) there were a significant number of cells containing multiple vesicles reminiscent of autophagic bodies. For *C. curvatus* fragments of pseudohyphae were observed mixed with elongated budding yeast cells (Figure 3). *C. terreus* exhibited a highly heterogeneous morphology with some cells being enlarged. These enlarged cells unlike typical Titan cells, were often not round, lacked single large vacuole, and often contained a large daughter cell (Figure 3). Such morphology suggested a possible failure in polarity establishment and a delay in the final cell separation during mitosis.

It was hypothetically possible that some of the non-*neoformans*/*gattii* species required longer incubation time to allow the development of Titan-like cells. To test this possibility, we also evaluated cell size and morphology after 5-days incubation under titanization conditions according to Dambuza et al., at 30°C. *M. furfur* was not included in this additional analysis. Although the mean cell diameter of *C. terreus* has further increased after 5 days of incubation under titanization conditions, no cells were larger than 10 microns (Figure 3 and S1). Interestingly, average cell diameter of *C. albidus* was smaller after 5 days of incubation in titanization conditions as compared to control, in contrast to cell enlargement observed after 48 h, even though one cell out of 100 had a diameter ~ 11 microns (Figure 3 and S1). No other tested species exhibited cell enlargement under these prolonged incubation conditions (Figure 3 and S1). While *C. albidus* strain MD22 consisted of mostly unbudded cells, the other strain of this species (MD41) revealed a heterogeneous population consisting of budding cells (Figure S1).

Titanization protocol developed by Trevijano-Contador et al. shares several aspects with the method introduced by Dambuza et al. [32,33]. Both protocols require the use of FBS and the incubation is performed at 37°C in the presence of 5% CO_2_. We initially tested if *C. neoformans* strain H99 forms robust Titans under conditions according to Trevijano-Contador et al. with the incubation temperature modified to 28°C and detected no cell enlargement after 18 hours (data not shown). In contrast to the above two protocols, the method described by Hommel et al. does not involve FBS, or 5% CO_2_, and the cells are incubated at 30°C [34]. In fact, FBS and incubation temperature of 37°C had a negative impact on titanization as described by Hommel et al. [34]. Furthermore, a lack of FBS in the method by Hommel et al. reduces the potential for variability associated with specific batches of the FBS. Therefore, *in vitro* titanization according to Hommel et al. constituted an ideal alternative method to be utilized in our study. We selected representative strains from each species (with the exception of *Malassezia*) and subjected these strains to the titanization protocol according to Hommel et al. (indicated in Figure 3, S1, and S2 as #4) [34]. A representative of *C. neoformans* (strain MD31) underwent titanization under these conditions although 5 days of incubation under conditions by Dambuza et al. has led to more robust titanization as compared to treatment according to Hommel et al. (Figure 3 and S1). The average cell diameter of 5 species (*C. aspenensis, C. curvatus, C. kuetzingii, C. laurentii, C. terrestris*) was smaller as compared to that of the control treatments and no cells with the diameter above 10 μm were observed (Figure 3, S1, and S2). Cell diameter of *C. albidus* and *S. cerevisiae* was not significantly different when compared to the control treatment (Figure 3). Average cell diameter of *C. terreus* was larger as compared to the control treatment, although 98% of the cells had a diameter of less than 10 microns (two cells out of 100 measuring ~10.5 and ~12.5 μm) (Figure 3 and S1). Furthermore, similar to the conditions by Dambuza et al., even the enlarged cells appeared different than typical Titan-like cells observed for *C. neoformans*, as no central enlarged vacuole was observed (Figure S1). Similarly, *C. uniguttulatus* underwent cell enlargement with 99% of the cells measuring no more than 10 μm (1 cell out of 100 had a diameter of ~11 μm) and the cell morphology did not suggest changes similar to typical titanization (Figure 3 and S1).

Our data suggested that *C. terreus*, unlike other tested species, undergoes morphological transition reminiscent of titanization, although the enlarged cells lacked the characteristic large vacuole. One of the other characteristics of the Titans described in *C. neoformans*/*C. gattii* species complex is an enlargement of the polysaccharide capsule [32,34]. We tested if the capsule has formed in any of the species subjected to titanization protocol according to Hommel et al. [34]. Consistent with previous findings, Titan cells of the *C. neoformans* (strain MD31) formed capsules (Figure S2(b)). Interestingly, *C. albidus* strain MD22 (and to lesser extent strain MD41), and *C. terrestris* (strains DS233 and DS290) formed capsules. The remaining species, including the *C. terreus* did not reveal capsule formation (Figure S2(b)).

In summary, our data suggest that representatives of the *C. neoformans*/*C. gattii* species complex are unique in their ability to form Titan cells when exposed to conditions described by Dambuza et al. with modified temperature to 30°C or under titanization conditions described by Hommel et al. [33,34].

## Discussion

The aim of this study was to assess the ability to titanize among various non-*C. neoformans*/non-*C. gattii* yeast species. We utilized two *in vitro* titanization protocols described recently by Dambuza et al. and Hommel et al. [33,34]. Knowing that representatives of *C. neoformans* and *C. gattii* species complex are unique within the order Tremellales for their ability to grow at a temperature above 30°C [43] and based on our tests (Table S1) it was necessary to slightly modify the protocol developed by Dambuza et al. by changing the incubation temperature from 37°C to 30°C. Consistent with previous findings [32,34], we demonstrated that *C. neoformans* strain H99 and at least 4 out of 7 strains of the *C. gattii* species complex were able to form Titan cells at 30°C in the presence of 5% CO_2_. Interestingly, *C. bacillisporus* strain B4546 exhibited particularly robust titanization. Hommel et al., have demonstrated that a truncated *PKR1* allele is associated with an increase in titanization [34]. It would be of interest to explore whether specific changes in gene expression and activity related to *PKR1* have led to increased titanization of the strain B4546. Dambuza et al. reported a lack of titanization in the *C. deuterogattii* strain R265 suggesting that perhaps under their specific conditions this strain is not stimulated to form Titan cells [33]. This was not the case in our study, as the R265 strain and at least 3 out of the remaining 6 strains of the *C. gattii* species complex tested here formed Titan cells under conditions similar to those utilized by Dambuza et al.

Our results clearly show that under *in vitro* conditions examined here neither of the evaluated 21 strains of non-*C. neoformans*/non-*C. gattii Cryptococcus* species is able to form *bona fide* Titan cells (Figure 3; Table 1, Figures S1, S2) as defined by two of the adopted criteria, namely cell body diameter of more than 10 μm and a large centrally positioned vacuole [33]. Changes at the micromorphology level were observed in the case of both tested strains of *C. curvatus*. While cultures incubated in YPD medium at 24°C consisted of only single, elongated budding cells, in the presence of 10% FBS in PBS, mycelial growth similar to species belonging to *Trichosporon* genus [44] were observed (Figure 3), to which *C. curvatus* is phylogenetically related [45]. Notably our results are consistent with the phylogenetic relatedness among tested species. Particularly striking were similar results obtained for *C. aspenensis, C. laurentii*, and *C. terrestris*, with all three species exhibiting a reduction in cell body diameter in titanization conditions. *C. terreus*, represented by one strain in our study, was an exception as we found occasional cells that were relatively larger than the rest of the population with the appearance reminiscent of the Titan-like cells. While these cells did not possess a large vacuole and were not larger than 12 μm, our findings suggest that *C. terreus* may also undergo morphological transition similar to titanization under specific conditions. Importantly, no capsule was detected even in enlarged cells of *C. terreus* under conditions by Hommel et al. [34], suggesting a lack of typical titanization. A lack of capsule in *C. terreus* was inconsistent with previous studies in this species, perhaps due to differences in the experimental approach [46]. *C. uniguttulatus* also revealed occasional enlarged cells under titanization conditions (Figure S1). However, in contrast to *C. terreus* most of these cells were not round and were indicative of failure in cell polarity and/or cell separation (Figure S1). Future studies should evaluate other characteristics associated with titanization in *C. terreus, C. uniguttulatus*, and other species tested here.

While the strains tested in our study represent various groups within basidiomycetous yeasts, it is still possible that other species may be capable of titanization. For instance more closely related *Cryptococcus* species *sensu stricto, C. amylolentus* may be able to titanize. Unfortunately, a limited number of isolates of this species is available, which undermines the significance of this potential test [47]. Additionally, it is plausible that some or all of the species tested in this study are capable of morphogenetic transition to form Titan-like cells but they may require specific environmental cue(s) not tested here. While this alternative remains a possibility, we would like to note that the formation of true hyphae, another morphological transition, is not common to all yeasts. Moreover, while *Candida albicans* forms enlarged “Goliath” cells in response to zinc limitation, this morphological transition is not conserved in other *Candida* spp., *Clavispora lusitaniae*, and *Debaryomyces hansenii* and Goliath cells are not morphologically similar to Titan cells [48].

The uniqueness of *C. neoformans*/*C. gattii* species complex for titanization under specific conditions described here emphasizes the success of these species to evolve as human pathogens. Future studies should reveal if other environmental cues exist to induce various yeast species to form Titan-like cells possessing all the characteristics described for *Cryptococcus* species complex. Several genes were recently described as implicated in titanization of *C. neoformans* [32–34]. It would be of interest to establish if these genes are conserved in species tested here, for instance in *C. terreus*, and other species outside of the *Cryptococcus* species complex. The growing number of sequenced fungal genomes, including the genomes of species belonging to the order Tremellales, will certainly augment studies towards elucidating the genetic basis of titanization.

## Supplementary Material

Supplemental Material
