# Supplementary material for: Titan cell formation is unique to Cryptococcus species complex
Source: Virulence. 2020 Jun 4;11(1):719–29. doi: 10.1080/21505594.2020.1772657 (PMC7549989; doi:10.1080/21505594.2020.1772657)
Supplement: Supplemental Material [file KVIR_A_1772657_SM8849.docx]

**Appendix 1**

**Table S1**. Strains used in this study. Their source, place of primary isolation, and actual taxonomic position are indicated^#^. Each strain has been tested for the ability to proliferate at 37°C.

| **Strain** | **Source^(1-5)^, primary isolation** | **Growth at 37**°**C** | **Ref** |
| --- | --- | --- | --- |
| *Cryptococcus albidus^a^*  MD22 | ^1^Interdigital spaces of the feet, component of the skin mycobiome (healthy male, Poland) | No | (1) |
| *Cryptococcus albidus^a^*  MD41 | ^1^Groin skin, component of the skin mycobiome (healthy female, Poland) | No | (1) |
| *Cryptococcus aspenensis^b^* , DS573 | ^2^*Populus tremuloides*, New York State (USA) | No | (2) |
| *Cryptococcus aspenensis^b^ ,* DS572 | ^2^*Populus tremuloides*, New York State (USA) | No | (2) |
| *Cryptococcus aspenensis^b^* , DS570 | ^2^*Populus tremuloides*, New York State (USA) | No | (2) |
| *Cryptococcus aspenensis^b^ ,* DS569 | ^2^*Populus tremuloides*, New York State (USA) | Yes | (2) |
| *Cryptococcus curvatus^c^* , MD110/2009 | ^1^Soil, Olsztyn, Poland | No | (1) |
| *Cryptococcus curvatus^c^ ,* MD120/2009 | ^1^Soil, Olsztyn, Poland | No | (1) |
| *C. deuterogattii^d^*  R265 (CBS10514) | ^3^Bronchial washings of infected patient from the Vancouver Island, British Columbia, Canada, VGIIa | Yes | (3) |
| *Cryptococcus bacillisporus*  WM161 | ^2^Environmental isolate, VGIIIa, San Diego CA, | Yes | (4) |
| *Cryptococcus bacillisporus*  CA1053 | ^2^Clinical isolate, VGIIIa, CA | Yes | (4) |
| *Cryptococcus bacillisporus*  B4546 | ^2^VGIIb | Yes | (4) |
| *Cryptococcus bacillisporus*  CA1508 | ^2^Clinical isolate, VGIIIb, CA | Yes | (4) |
| *Cryptococcus bacillisporus*  NIH312/ATCC34880 | ^2^Clinical isolate from cerebrospinal fluid, VGIIIb | Yes | (5) |
| *Cryptococcus tetragattii*  MMRL2651 | ^2^Clinical isolate, VGIV, Botswana | Yes | (5) |
| *Cryptococcus kuetzingii^a*^* MUCL27749 | ^2^Intestinal tract, *Blatta orientalis* | No | (1) |
| *Cryptococcus laurentii^e^* , DS620 | ^2^*Picea abies*, New York State (USA) | Yes | (1) |
| *Cryptococcus laurentii^e^* , DS619 | ^2^*Picea abies*, New York State (USA) | Yes | (1) |
| *Cryptococcus laurentii^e^* , DS621 | ^2^*Picea abies*, New York State (USA) | Yes | (1) |
| *Cryptococcus laurentii^e^* , DS288 | ^2^*Colophospermum mopane*, Botswana | Yes | (1) |
| *Cryptococcus laurentii^e^* , DS386 | ^2^*Colophospermum mopane*, Botswana | Yes | (1) |
| *Cryptococcus neoformans^f^*  H99 (var. *grubii)* (ATCC 208821) | ^2^Male patient with Hodgkin's disease, New York, USA | Yes | - |
| *Cryptococcus neoformans^f^*  MD31 (var. *grubii*) | ^1^Bronchoalveolar lavage (BAL) of patient with AIDS, Poland | Yes | - |
| *Cryptococcus terrestris^g^* , DS291 | ^2^*Colophospermum mopane*, Botswana | No | (6) |
| *Cryptococcus terrestris^g^ ,* DS233 | ^2^*Colophospermum mopane*, Botswana | No | (6) |
| *Cryptococcus terrestris^g^ ,* DS234 | ^2^*Colophospermum mopane*, Botswana | No | (6) |
| *Cryptococcus terrestris^g^ ,* DS290 | ^2^*Colophospermum mopane*, Botswana | No | (6) |
| *Cryptococcus terreus^h^* , DUMC177.8 | ^2^Soil, New Zealand | No | (5) |
| *Cryptococcus uniguttulatus^i^* , MD29 | ^1^Interdigital spaces of the feet, component of the skin mycobiome (healthy female, Poland) | No | - |
| *Cryptococcus uniguttulatus^i^* , MD26 | ^1^Soil, Wroclaw, Poland | No | - |
| *Malassezia furfur^j^* , CBS7019 | ^4^Skin of 15-year-old girl with p*ityriasis versicolor*, Finland | Yes | - |
| *Malassezia sympodialis^k^*  ATCC42132 | ^4^Skin of a male with pityriasis versicolor | Yes | - |
| *Saccharomyces cerevisiae^l^* BY4741 | ^5^Euroscarf, Germany (isogenic to S288C) | Yes | (7) |

^#^ source: <http://www.indexfungorum.org/names/names.asp>

Source of strains: ^1^L. Kozubowski collection, Clemson University, Clemson, SC, USA; ^2^kindly provided by J. Heitman, Duke University, Durham, NC, USA; ^3^gift from H. de Cock, Utrecht University, Utrecht, The Netherlands; ^4^obtained from Westerdijk Institute, Utrecht, The Netherlands; ^5^obtained from Euroscarf, Frankfurt, Germany;

Actual taxonomic position: **^a^***Naganishia albida*; **^b^***Papiliotrema aspenensis;* **^c^***Cutaneotrichosporon curvatum;* **^d^***Cryptococcus gattii;* **^e^***Papiliotrema laurentii;* ***^f^****Cryptococcus neoformans;* ***^g^****Papiliotrema terrestris;* ***^h^****Solicoccozyma terrea;* ***^i^****Filobasidium uniguttulatum;* ***^j^****Malassezia furfur;* ***^k^****Malassezia sympodialis;* ***^l^****Saccharomyces cerevisiae;* *also known as *Cryptococcus albidus* var. *kuetzingii* (Fell & Phaff) Fonseca, Scorzetti & Fell.

**SUPPLEMENTARY FIGURES**


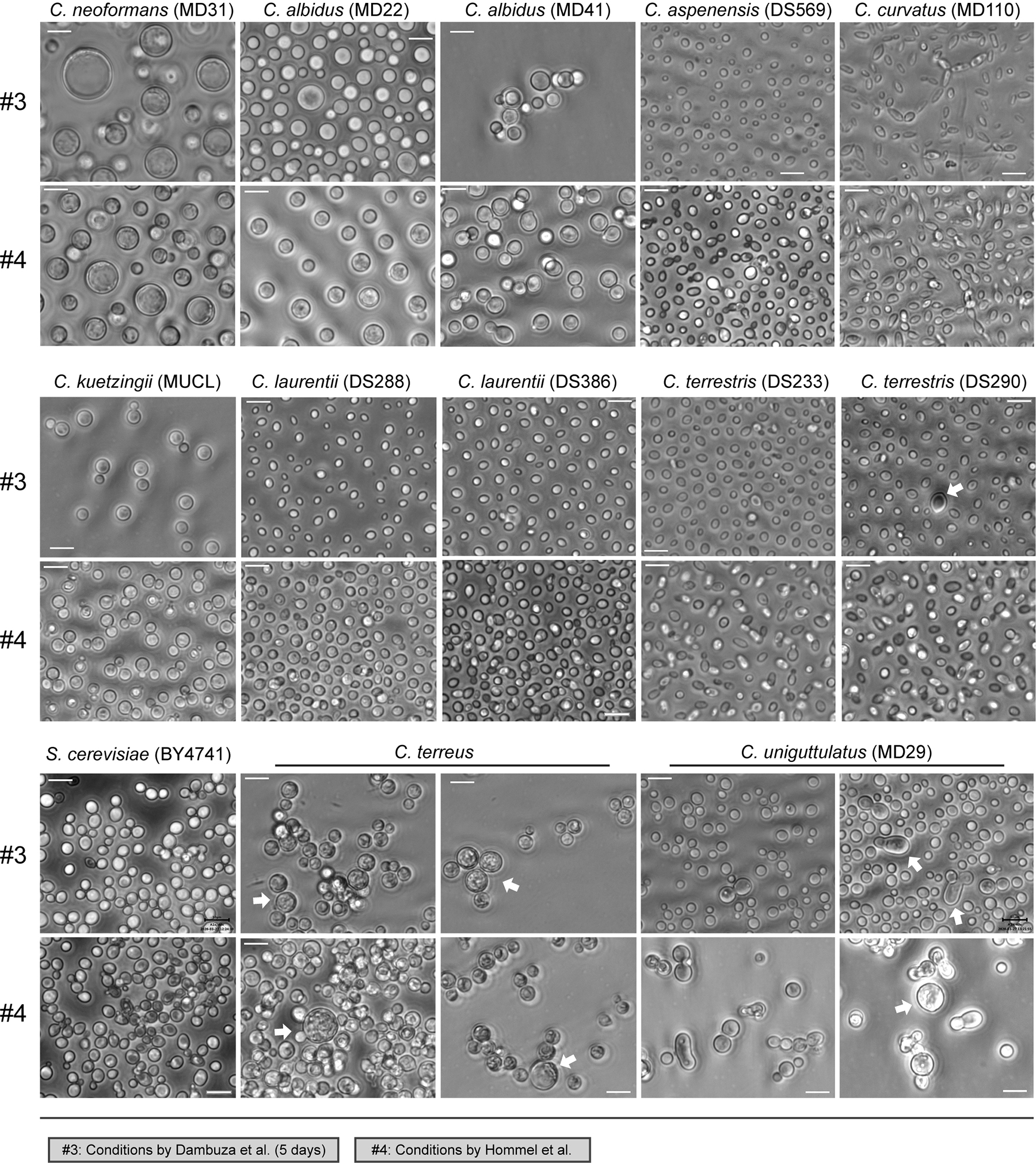


**Figure S1. Members of the *Cryptococcus* species complex are unique in their ability to form Titan cells**

*C. neoformans* (MD31), and representatives of other species, as indicated, were subject to the *in vitro* titanization protocol established by Dambuza et al. (8) with a modified temperature to 30°C and incubated for 5 days (indicated as #3) or subject to conditions developed by Hommel et al. (9)(indicated as #4). Arrows point to cells that underwent enlargement and are reminiscent of Titan-like cells. Bars represent 10 µm.


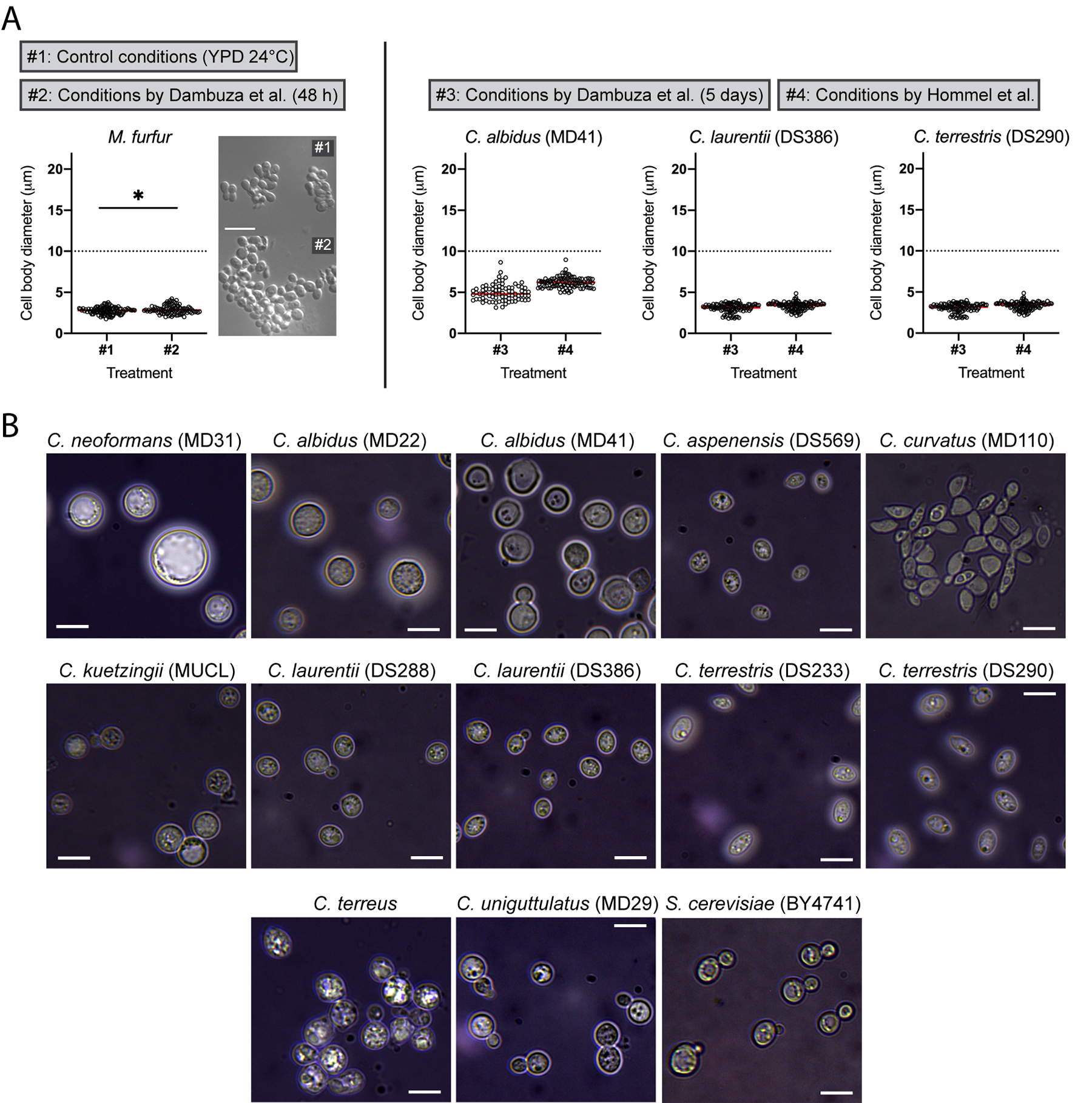


**Figure S2. Members of the *Cryptococcus* species complex are unique in their ability to form Titan cells**

A. M. furfur was grown in YPD control medium at 24°C (indicated as #1), or subject to the *in vitro* titanization protocol established by Dambuza et al. (8) with a modified temperature to 30°C and incubated for 48 h (indicated as #2). Indicated other species were subject to the *in vitro* titanization protocol established by Dambuza et al. with a modified temperature to 30°C and incubated for 5 days (indicated as #3) or subject to conditions developed by Hommel et al. (9)(indicated as #4). B. Indicated species were stained with nigrosin to detect capsule formation. Bars represent 10 µm.

**References**

1. Liu XZ, Wang QM, Goker M, Groenewald M, Kachalkin AV, Lumbsch HT, et al. Towards an integrated phylogenetic classification of the Tremellomycetes. Stud Mycol. 2015;81:85-147.

2. Ferreira-Paim K, Ferreira TB, Andrade-Silva L, Mora DJ, Springer DJ, Heitman J, et al. Phylogenetic analysis of phenotypically characterized *Cryptococcus laurentii* isolates reveals high frequency of cryptic species. PLoS ONE. 2014;9(9):e108633.

3. Kidd SE, Hagen F, Tscharke RL, Huynh M, Bartlett KH, Fyfe M, et al. A rare genotype of *Cryptococcus gattii* caused the cryptococcosis outbreak on Vancouver Island (British Columbia, Canada). Proceedings of the National Academy of Sciences of the United States of America. 2004;101(49):17258-63.

4. Springer DJ, Billmyre RB, Filler EE, Voelz K, Pursall R, Mieczkowski PA, et al. *Cryptococcus gattii* VGIII isolates causing infections in HIV/AIDS patients in Southern California: identification of the local environmental source as arboreal. PLoS pathogens. 2014;10(8):e1004285.

5. Fernandes KE, Dwyer C, Campbell LT, Carter DA. Species in the *Cryptococcus gattii* Complex Differ in Capsule and Cell Size following Growth under Capsule-Inducing Conditions. mSphere. 2016;1(6).

6. Litvintseva AP, Carbone I, Rossouw J, Thakur R, Govender NP, Mitchell TG. Evidence that the human pathogenic fungus *Cryptococcus neoformans* var. *grubii* may have evolved in Africa. PLoS ONE. 2011;6(5):e19688.

7. Brachmann CB, Davies A, Cost GJ, Caputo E, Li J, Hieter P, et al. Designer deletion strains derived from *Saccharomyces cerevisiae* S288C: a useful set of strains and plasmids for PCR-mediated gene disruption and other applications. Yeast (Chichester, England). 1998;14(2):115-32.

8. Dambuza IM, Drake T, Chapuis A, Zhou X, Correia J, Taylor-Smith L, et al. The *Cryptococcus neoformans* Titan cell is an inducible and regulated morphotype underlying pathogenesis. PLoS pathogens. 2018;14(5):e1006978.

9. Hommel B, Mukaremera L, Cordero RJB, Coelho C, Desjardins CA, Sturny-Leclere A, et al. Titan cells formation in *Cryptococcus neoformans* is finely tuned by environmental conditions and modulated by positive and negative genetic regulators. PLoS pathogens. 2018;14(5):e1006982.

ITS sequences used for phylogenetic tree construction (obtained from the GenBank of the National Center for Biotechnology Information (NCBI, Bethesda, MD, USA).

**GenBank accession number (red font)**

>Cryptococcus_gattii_VGI **KY102792.1**

TACACACCGCCGTCGCTACTACCGATTGAATGGCTTAGTGAGATCTCTGGATTGGCGTTGGGGAGCCGGCAACGGCACCCCTTGGCTGAGAAGCTGATCAAACTTGGTCATTTAGAGGAAGTAAAAGTCGTAACAAGGTTTCCGTAGGTGAACCTGCGGAAGGATCAGTAGAGAATGCTGGGCTTCGGTCCATTTATCTACCCATCTACACCTGTGAACTGTTTATGTGCTTCGGCACGTTTTACACAAACTTCTAAATGTAATGAATGTAATCTTATTATAACAATAATAAAACTTTCAACAACGGATCTCTTGGCTTCCACATCGATGAAGAACGCAGCGAAATGCGATAAGTAATGTGAATTGCAGAATTCAGTGAATCATCGAGTCTTTGAACGCAACTTGCGCCCTTTGGTATTCCGAAGGGCATGCCTGTTTGAGAGTCATGAAAATCTCAATCCCTCGGGTTTTATTACCTGTTGGACTTGGATTTGGGTGTTTGCCGCGACCTGCAAAGGACGTCGGCTCGCCTTAAATGTGTTAGTGGGAAGGTGATTACCTGTCAGCCCGGCGTAATAAGTTTCGCTGGGCCTATGGGGTAGTCTTCGGCTTGCTGATAACAACCATCTCTTTTTTGTTTGACCTCAAATCAGGTAGGGCTACCCGCTGAACTTAAGCATATCAA

>Cryptococcus_bacillisporus_WM161 **KY102615.1**

TCGTAACAAGGTTTCCGTAGGTGAACCTGCGGAAGGATCAGTAGAGAATACTGGACTTCGGTCCATTTATCTACCCATCTACACCTGTGAACCGTTTATGTGCTTCGGCACGTTTTACACAAACTTCTAAATGTAATGAATGTAATCTTATTATAACAATAATAAAACTTTCAACAACGGATCTCTTGGCTTCCACATCGATGAAGAGCGCAGCGAAATGCGATAAGTAATGTGAATTGCAGAATTCAGTGAATCATCGAGTCTTTGAACGCAACTTGCGCCCTTTGGTATTCCGAAGGGCATGCCTGTTTGAGAGTCATGAAAATCTCAATCCCTCGGGTTTTATTACCTGTTGGACTTGGATTTGGGTGTTTGCCGCGACCTGGAAAGGACGTCGGCTCGCCTTAAATGTGTTAGTGGGAAGGTGATTACCTGTCAGCCCGGCGTAATAAGTTTCGCTGGGCCTATGGGGTAGTCTTCGGCTTGCTGATAACAACCATCTCTTTTTGTTGACCTCAAATCAGGTAGGGCTACCCGCTGAACTTAAGCATATCAATAAGCGGA

>Cryptococcus_deuterogattii_R265 **KY102661.1**

GTCGTAACAAGGTTTCCGTAGGTGAACCTGCGGAAGGATCAGTAGAGAATACTGGACTTCGGTCCATTTATCTACCCATCTACACCTGTGAACTGTTTATGTGCTTCGGCACGTTTTACACAAACTTCTAAATGTAATGAATGTAATCTTATTATAACAATAATAAAACTTTCAACAACGGATCTCTTGGCTTCCACATCGATGAAGAACGCAGCGAAATGCGATAAGTAATGTGAATTGCAGAATTCAGTGAATCATCGAGTCTTTGAACGCAACTTGCGCCCTTTGGTATTCCGAAGGGCATGCCTGTTTGAGAGTCATGAAAATCTCAATCCCTCAGGTTTTATTACCTGTTGGACTTGGATTTGGGTGTTTGCCGCGACCTGCAAAGGACGTCGGCTCGCCTTAAATGTGTTAGTGGGAAGGTGATTACCTGTCAGCCCGGCGTAATAAGTTTCGCTGGGCCTATGGGGTAGTCTTCGGCTTGCTGATAACAACCATCTCTTTTTGTTTGACCTCAAATCAGGTAGGGCTACCCGCTGAACTTAAGCATATCAA

>Cryptococcus_uniguttulatus_CBS4257 **KY103443.1**

TAACAAGGTTTCCGTAGGTGAACCTGCGGAAGGATCATTATTGAATTTAGTTGTCTGGCTTTCGCCGACGACGATATCATTATCCATAACACCTGTGCACTGTTGGATGTTTAATACATCCGTTTTACACTAAACAATATTGTTACAAATGTAGTCTTATTATAACATAATAAAACTTTCAACAACGGATCTCTTGGCTCTCGCATCGATGAAGAACGCAGCGAAATGCGATAAGTAATGTGAATTGCAGAATTCAGTGAATCATCGAATCTTTGAACGCACCTTGCGCTCCTTGGTATTCCGAGGAGCATGCCTGTTTGAGTGTCATGAAACTCTCAAACTCTTGTTTTGGATGCAAATCCTTGCTTGAGTTTGGACTTGGGTGTTTGCCGGTGATGAACCGACTCGCCTTAAACATATTAGCTGGACTTGTCTATATGACTGGTTTGACTTGGCATAATAAGTATTTTGCTAAGGACATCTTCGGATGGCCAGTACCTAGGCTCTGTGTCTGCTAACTAAACCATCACTTGGAGTGCATCTTTATGGTGTTGCTTCCTGTGTATACTTTGACATCTGACCTCAAATCAGGTAGGACTACCCGCTGAACTTAAGCATATCAA

>Cryptococcus_neoformans_H99 **KU729082.1**

GGTTTCCGTAGGTGAACCTGCGGAAGGATCAGTAGAGAATATTGGACTTCGGTCCATTTATCTACCCATCTACACCTGTGAACTGTTTATGTGCTTCGGCACGTTTTACACAAACTTCTAAATGTAATGAATGTAATCTTATTATAACAATAATAAAACTTTCAACAACGGATCTCTTGGCTTCCACATCGATGAAGAACGCAGCGAAATGCGATAAGTAATGTGAATTGCAGAATTCAGTGAATCATCGAATCTTTGAACGCAACTTGCGCCCTTTGGTATTCCGAAGGGCATGCCTGTTTGAGAGTCATGAAAATCTCAATCCCTCGGGTTTTATTACCTGTTGGACTTGGATTTGGGTGTTTGCCGCGACCTGCAAAGGACGTCGGCTCGCCTTAAATGTGTTAGTGGGAAGGTGATTACCTGTCAGCCCGGCGTAATAAGTTTCGCTGGGCCTATGGGGTAGTCTTCGGCTTGCTGATAACAACCATCTCTTTTTGTTTGACCTCAAATCAGGTAGGGCTACCCGCTGAACTTAAGCATATCAATAA

>Cryptococcus_albidus_CBS2824 **KY102588.1**

GTAACAAGGTTTCCGTAGGTGAACCTGCGGAAGGATCATTAATGATTAACTGTCTGTCGAGCTTGCTCACAGACTTATCATATCCATAACACCTGTGCACTTGTCGGATGGCTTAGTGAAGACCGCAAGGTTGGATCTATCCATCTACTTTACATAACAATTTAGTAACAAATGTAGTCTTATTATAACATAATAAAACTTTCAACAACGGATCTCTTGGCTCTCGCATCGATGAAGAACGCAGCGAAATGCGATAAGTAATGTGAATTGCAGAATTCAGTGAATCATCGAATCTTTGAACGCACCTTGCGCTCCCTGGTATTCCGGGGAGCATGCCTGTTTGAGTGTCATGAAAACCCTCAACCTTAGATTGGTTAACACCTTTCTTTGGCTTGGATTTGGACGTTTGCCGATGATAAGTCGGCTCGTCTTAAAAGTAATAGCTGGATCTGTCTCGCGACATGGTTTGACTTGGCGTAATAAGTATTTCGCTAAGGACATCTTCGGATGGCCGCGTTGCAAGACTAAAGACCGCTTTCTAATCCATTGATCTTCGGATTAATATTCTTGACATCTGGCCTCAAATCAGGTAGGACTACCCGCTGAACTTAAGCATATCAA

>Cryptococcus_deneoformans_CBS7822 **KY102624.1**

AACAAGGTTTCCGTAGGTGAACCTGCGGAAGGATCATTAGAGAATATTGGACTTTGGTCCATTTATCTACCCATCTACACCTGTGAACTGTTTATGTGCTTCGGCACGTTTTACACAAACTTCTAAATGTAATGAATGTAATCATATTATAACAATAATAAAACTTTCAACAACGGATCTCTTGGCTTCCACATCGATGAAGAACGCAGCGAAATGCGATAAGTAATGTGAATTGCAGAATTCAGTGAATCATCGAGTCTTTGAACGCAACTTGCGCCCTTTGGTATTCCGAAGGGCATGCCTGTTTGAGAGTCATGAAAATCTCAATCCCTCGGGTTTTATTACCTGTTGGACTTGGATTTGGGTGTTTGCCGCGACCTGCAAAGGACGTCGGCTCGCCTTAAATGTGTTAGTGGGAAGGTGATTACCTGTCAGCCCGGCGTAATAAGTTTCGCTGGGCCTATGGGGTAGTCTTCGGCTTGCTGATAACAACCATCTCTTTTTGTTTGACCTCAAATCAGGTAGGGCTACCCGCTGAACTTAAGCATATCAATAAGCGGA

>Cryptococcus_aspenensis_DS569 **KC485497.1**

TCATCTCTATATCCCTCAACCTCTGTGAACCGTGGACCCTCGGGTCTATTTTACAAACATCAGTGTAATGAACGTAATTATCATAACAAAAACAAAACTTTCAACAACGGATCTCTTGGCTCTCGCATCGATGAAGAACGCAGCGAAATGCGATAAGTAATGTGAATTGCAGAATTCAGTGAATCATCGAATCTTTGAACGCACCTTGCGCCTCTCGGTATTCCGAGAGGCATGCCTGTTTGAGTGTCATGAAATCTCAATCCCCTCGGGTTTTAGGACCCGGGTCGGACTTGGACATGGGCGTCTGCCGGTTAAACGGCTCGCCTCAAATGACTTAGTGGATCTCTCAGCATCCGTGACAGACGTAATAAGTTTCGTCTTGTCCCTTGCAGATGAGTCCGCTCACAACCTGCCATCGCGCACTTTTAGACTCTGACCTCAAATCAGGTAGGACTACCCGCTGAACTTAAGCATATCA

>Cryptococcus_terrestris_DS290 **KC485479.1**

TACCACCTCTGTGAACCGTTGACCTCCGGGTTAATAATCAAACATCAGTGTAACGAACGTAAGAGTATCTTAACGAAACAAAACTTTCAACAACGGATCTCTTGGCTCTCGCATCGATGAAGAACGCAGCGAAATGCGATAAGTAATGTGAATTGCAGAATTCAGTGAATCATCGAATCTTTGAACGCACCTTGCGCCTTTTGGTATTCCGAAAGGCATGCCTGTTTCAGTGTCATGAAATCTCAATCTAATATGTTTTCTGAACATGTTAGACTTGGACTTGGGCGTCTGCCAGTGATGGCTCGCCTCAAATGACTTAGTGGAACATCCCACATCAGTGTTAGACGTAATAAGTTTCGTCTCTCCTTGTGGTGATGACTGCTCAGAACCTGCCATCGCGCACTTTTGACTTTGACCTGAAATCAGGTAGGGCTACCCGCTGAACTTAAGCATATCAATA

>Cryptococcus_laurentii_DS288 **KC485478.1**

TTATCTCTATATCCCTCACCTCTGTGAACTGTGGACCTCCGGGTCTATTTAACAAACATCAGTGTAATGAACGTATATATCATTAAACAAAACAAAACTTTCAACAACGGATCTCTTGGCTCTCGCATCGATGAAGAACGCAGCGAAATGCGATAAGTAATGTGAATTGCAGAATTCAGTGAATCATCGAATCTTTGAACGCACCTTGCGCCTTTTGGTATTCCGAAAGGCATGCCTGTTTGAGTGTCATGAAATCTCAATCCCCCTGGGTTTATGATCTGGGTCGGACTTGGATATGGGCGTCTGCCGGTCACACGGCTCGCCTCAAATGACTTAGTGGATCTCTCTGCATCCGTGACAGACGTAATAAGTTTCGTCTTGTCCCTTGCTTATGAGTCTGCTCATAACCTGCCATCGCGCACTTTTAGACTCTGACCTCAAATCAGGTAGGACTACCCGCTGAACTTAAGCATATCAATAA

>Cryptococcus_terreus_DUMC177.8 **KY102950.1**

ACAAGGTTTCCGTAGGTGAACCTGCGGAAGGATCATTAGTGAATTAAACATGCTTGGTGTCTTCGCTTCGGCAAGGCCCTTGCTTAAATCACATCCTAACACCTGTGAACTGTAAGACGTATGATGAGGTCTTTGGCCAAGTCATCGTCTGCCCATTTTTAACAAACAATTAATGTAACAAACGTAGTCTTATTATAACCTAATAAAACTTTCAACAACGGATCTCTTGGCTCTCGCATCGATGAAGAACGCAGCGAAATGCGATAAGTAATGTGAATTGCAGAATTCAGTGAATCATCGAATCTTTGAACGCACCTTGCGCTCTTTGGTATTCCGAAGAGCATGCCTGTTTGAGTGTCATGAAAATATCAACCTTGACTTGGGTTTTGTGCTCTAGTCTTGGCTTGGAATTGGGTGCTTGCCGCTTTTACGAGCGGCTCACCTTAAATGTATTAGCTGGATCTGTCTTTGAGACTTGGTTTGACTTGGCGTAATAAGTATTTCGCTAAGGACATTCTTCGGAGGGGCCTCGTTTCTAGGACGCTTGTCCGCTTCCCAATACAAGTTCCACCTCGTGGACATGACTTTTTATTATCTGGCCTCAAATCAGGTAGGACTACCCGCTGAACTTAAGCATATCAA

>Cryptococcus_kuetzingii_CBS1926 **AF145327.2**

TCCGTAGGTGAACCTGCGGAAGGATCATTAATGATTGACCGTCTGTCGAGCTTGCTCACAGGCACATCATATCCATAACACCTGTGCACTTGTCGGATGGCTTAGTGAAGACCGCAAGGTTGGATCTATCCATCTACTTTACATAACAATTCTGTAACAAATGTAGTCTTATTATAACATAATAAAACTTTCAACAACGGATCTCTTGGCTCTCGCATCGATGAAGAACGCAGCGAAATGCGATAAGTAATGTGAATTGCAGAATTCAGTGAATCATCGAATCTTTGAACGCACCTTGCGCTCCTTGGTATTCCGAGGAGCATGCCTGTTTGAGTGTCATGAAAACCCTCAACCCTAGATTGGTTAAAACCTTTCTTTGGTTTGGATTTGGACGTTTGCCGATGATAAGTCGGCTCGTCTTAAAAGTAATAGCTGGATCTGTCTCGCGACATGGTTTGACTTGGCGTAATAAGTATTTCGCTAAGGACATCTTCGGATGGCCGCGTTGCAGGACTAAAGACCGCTTTCTAATCCATTGATCTTCGGATTAATACTCTTGACATCTGGCCTCAAATCAGGTAGGACTACCCGCTGAACTTAAGCATATCAATAAGCGGAGGA

>Cryptococcus_curvatus_ATCC10567 **EU266558.1**

AATATGACGCAGCCATCACAGGTCACATTGATATGATATAGCCCGCCCGCAGATCATCTAATGATCTTTCCGTAGGTGAACCTGCGGAAGGATCATTAGTGATTTGCCTTCGGGCTAAACTATATCCATAACACCTGTGAACTGTTGATTGACTTCGGTCAATATTTTTACAAACATTGTGTAATGAACGTCATGTTATAATAACAAATATAACTTTCAACAACGGATCTCTTGGCTCTCGCATCGATGAAGAACGCAGCGAAATGCGATAAGTAATGTGAATTGCAGAATTCAGTGAATCATCGAATCTTTGAACGCAACTTGCGCTCTCTGGTATTCCGGAGAGCATGCCTGTTTGAGTGTCATGAAATCTCAACCATTAGGGTTTCTTAATGGCTTGGATTTGGACGTTTGCCAGTCAAATGGCTCGTCTTAAAAGAGTTAGTGAATTTAACATTTGTCTTCTGGCGTAATAAGTTTCGCTGGGCTGATAGTGTGAAGTTTGCTTCTAATCGTCCGCAAGGACAATTCTTGAACTCTGGCCTCAAATCAGGTAGGACTACCCGCTGAACTTAAGCATATCAATAAGCGGAGAGAATCATTAGTGATTTGCCTTCCGGCTAAACTATATTCATAAC

>Cryptococcus_tetragattii_VGIV **KY102969.1**

TCGTAACAAGGTTTCCGTAGGTGAACCTGCGGAAGGATCAGTAGAGAATACTGGACTTCGGTCCATTTATCTACCCATCTACACCTGTGAACTGTTTATGTGCTTCGGCACGTTTTACACAAACTTCTAAATGTAATGAATGTAATCTTATTATAACAATAATAAAACTTTCAACAACGGATCTCTTGGCTTCCACATCGATGAAGAACGCAGCGAAATGCGATAAGTAATGTGAATTGCAGAATTCAGTGAATCATCGAGTCTTTGAACGCAACTTGCGCCCTTTGGTATTCCGAAGGGCATGCCTGTTTGAGAGTCATGAAAATCTCAATCCCTCGGGTTTTATTACCTGTTGGACTTGGATTTGGGTGTTTGCCGCGACCTGCAAAGGACGTCGGCTCGCCTTAAATGTGTTAGTGGGAAGGTGATTACCTGTCAGCCCGGCGTAATAAGTTTCGCTGGGCCTATGGGGTAGTCTTCGGCTTGCTGATAACAACCATCTCTTTTTTGTTGACCTCAAATCAGGTAGGGCTACCCGCTGAACTTAAGCATATCAATAAGCGGA

>Malassezia_sympodialis_CBS7222 **KY104180.1**

TGATGGCTTAGTGAGCCTTTGGGATCGGCAGCTTGGACCGTCACCGGCCACATTAGACCGCTGAAAACTTATGCAAACTTGGTCATTTAGAGGAAGTAAAAGTCGTAACAAGGTTTCTGTAGGTGAACCTGCAGAAGGATCATTAGTGAAAGTTTCGGGCCTGCCATACGGACGCAAACACGTCTCTGGCGCCCATCACTATATCCATACCAACCCCTGTGCACTGTGATGACGAATGTCATCGAACAAAAAAAACTCGTATGGTTGAATGTACGTGAAATTGTAGGTATAGCCTACGAACTATACACAACTTTCGACAACGGATCTCTTGGTTCTCCCATCGATGAAGAACGCAGCGAAACGCGATAGGTAATGTGAATTGCAGAATTCCGTGAATCATCGAATCTTTGAACGCACCTTGCGCTCCATGGTATTCCGTGGAGCATGCCTGTTTGAGTGCCGCGAATTCTCCCTCCCCTTACGGTGGCCGAAAGGCCGAAGTAGGGCGGACGGGGTAGGATGGGTGTTGCTGCCTGGGGATTGTACCAGGCTCGCCCGAAATGCATAAGCGCCAGGACCCTCGCTACCGCTCTCTAGGGAAGAGTGGCTAAGCGACCGCTGAGCATGGCATGATACGTCATTTGCTGTGTGTGGGCGAACGGGTTGGAGAGGTGTCTGCTTTACCAGCCCTTTTTAATTC

>Malassezia_furfur_CBS7019 **AY743635.1**

GATCATTAGTGAAAGCAAGGGCCAGCCATACGGACGGCGCTACTCGCGTACAACGTCTCTGGCGCCCAACTTTACACAATATCCCACAAACCCGTGTGCACCGTTTGGATGAGTTGGACCTCGCAAGAGGCCTGGCTCTCCAATCCATTTCTACCAAACTCGTATGGTTGTATGAACGTGGAAATCGTTGGACCGTAACTGGCCAACAACCAATAATACAACTTTCGACAACGGATCTCTTGGTTCTCCCATCGATGAAGAACGCAGCGAAACGCGATAGGTAATGTGAATTGCAGAATTCCGTGAATCATCGAATCTTTGAACGCACCTTGCGCTCCATGGTATTCCGTGGAGCATGCCTGTTTGAGTGCCGTGAATTCTCTCTCCCCAAGCGGTTGCGATTGCACTGCTTTGGCGGACGAGGTTGGATGGGTGCTTCTGCCTGTTTCGCAAGAAACAGGCTCGCCCGAAATGCATTAGCGCCTTTGGGACACACTCTGCAAACCGCTCTGAAAGGGAGGGCGGCAGAGGGGATGGAGGAACTCCGCCCGTCAGCTATACCAAACTTTGCCCCAGGCAAGCGCATGGCATGATACGTCATTTGCTGTGTGTGCGTCCTGAGGCAAGGGGCCGATGTGGTGCCTTTGTCACTCTGTGGGTGTGTTGGTGCGCTACCAATCCTGCGTGGAGGCTGGTCTGTGCGCAAGCATGGAGCAGTTCTTGTGAACGCATTCCCTTTTTCATTTCTGGTCTCAAATCAGGTAGGATCACCCGCTGAACTTAAGCATATCAATAAGCGGGAGGA
